# Increased *MLH1*, *MGMT*, and *p16INK4a* methylation levels in colon mucosa potentially useful as early risk marker of colon cancer

**DOI:** 10.1080/23723556.2025.2503069

**Published:** 2025-05-10

**Authors:** Yvonne Wettergren, Peter Rolny, Helena Lindegren, Elisabeth Odin, Victoria Rotter Sopasakis, Simon Keane, Katarina Ejeskär

**Affiliations:** a Department of Surgery, Institute of Clinical Sciences, Sahlgrenska Academy, University of Gothenburg, Gothenburg, Sweden; b Department of Surgery, Region Västra Götaland, Sahlgrenska University Hospital, Gothenburg, Sweden; c Department of Molecular and Clinical Medicine, Institute of Medicine, Sahlgrenska Academy, University of Gothenburg, Gothenburg, Sweden; d Department of Medicine, Division of Gastroenterology and Hepatology, Region Västra Götaland, Sahlgrenska University Hospital/Östra, Gothenburg, Sweden; e Department of Clinical Chemistry, Sahlgrenska University Hospital, Gothenburg, Sweden; f Department of Laboratory Medicine, Institute of Biomedicine, Sahlgrenska Academy, University of Gothenburg, Gothenburg, Sweden; g Translational Medicine, School of Health Sciences, University of Skövde, Skövde, Sweden

**Keywords:** Colon cancer, normal mucosa, methylation; MLH1, MGMT, CDKN2a, LINE-1, gene expression, MSI, rs3814960

## Abstract

The genes MutL Homolog 1 (*MLH1*), O6-methylguanine-DNA methyltransferase (*MGMT*), and cyclin-dependent kinase inhibitor *p16INK4a* are commonly downregulated by hypermethylation in colorectal cancer. Long interspersed nucleotide element 1 (LINE-1) can be used as marker for global hypomethylation. This study compared *MLH1*, *MGMT*, *p16INK4a*, and LINE-1 methylation with gene expression in colon tumors, matched non-cancerous mucosa, and control mucosa to identify signs of premalignancy. Tissues were obtained from 20 colon cancer patients and 40 controls. CpG site methylation was quantified by pyrosequencing, expression by qPCR, and MSI/*KRAS* status by fragment analysis and droplet digital PCR. *MLH1*, *MGMT*, and *p16INK4a* methylation was increasingly higher in control mucosa, non-cancerous mucosa, and tumors. *MLH1* expression was lower in tumors compared to non-cancerous mucosa but higher compared to control mucosa. Tumoral LINE-1 methylation correlated negatively with *MLH1* (*r* = −0.51, *p* = .021) and p16INK4a (*r* = −0.55, *p* = .012) methylation, but positively (*r* = 0.74, *p* = .0002) with *MLH1* expression. A *p16INK4a* SNP (rs3814960 C>T) was associated with methylation, expression, and MSI/*KRAS* status. Aberrant methylation of tumor suppressor genes in colon mucosa could be an early cancer risk marker. Control mucosa is a more reliable reference than non-cancerous mucosa when identifying premalignant changes. Extended studies will evaluate the possible association between rs3814960 and cancer susceptibility.

**Trial registration**: NCT03072641

## Introduction

Epigenetic alterations are considered to be early changes in the development of colorectal cancer. These alterations include methylation of CpG sites in the promoter region or first exons of tumor suppressor genes leading to transcriptional silencing.^1^ High levels of DNA methylation in tumor suppressor genes may be associated with a positive CpG island methylator phenotype (CIMP^+^) that can be either cancer-associated or age-related.^2^ CIMP^+^ is often found in tumors with microsatellite instability (MSI) caused by inactivation of the mismatch repair gene MutL Homolog 1 (*MLH1)*. The inactivation often occurs through methylation of the *MLH1* gene promoter. The *O*^6^-methylguanine-DNA methyltransferase (*MGMT*) gene encodes a protein involved in repair of DNA adducts and is frequently inactivated by methylation of the promoter region or exon 1. Hypermethylation of *MGMT* leads to G>A mutations in the *KRAS* gene.^3^ Previous results suggest that the methylation patterns of *MGMT* and *MLH1* are mutually exclusive in colorectal cancer.^4,5^

Another region commonly hypermethylated in colorectal cancer lies within the INK4b-ARF-INK4a locus. This locus encodes CDKN2b/p15INK4b and CDKN2a/p16INK4a, two cyclin-dependent kinase inhibitors that regulate the G1 phase of the cell cycle and an unrelated protein called CDKN2a/p14ARF (alternative reading frame) which regulates cell cycle arrest and apoptosis by both p53-dependent and independent pathways.^6^ Two different expression patterns of *p16INK4a* have been described in the progression from normal to malignant tissue.^7^ One is associated with oncogene-induced senescence whereby the cells are protected from various types of stressors. In this case, *p16INK4a* is overexpressed in benign and pre-malignant tissue but downregulated in malignant tumors. In the other pattern, the gene is overexpressed also in malignant tissue due to alterations in the Rb pathway. Methylation of CpG sites in the promoter region or in the first and second exon of *p16INK4a* has been shown to be critical for transcriptional silencing, which is an important mechanism in colorectal carcinogenesis.^8^

It has been suggested that epigenetic changes in the mucosa surrounding the tumor may occur before onset of genetic alterations in the early phase of carcinogenesis.^9^ Aberrant methylation in mucosa distant from tumor seems to be associated with the prognosis of patients with colorectal cancer. In a previous study, we showed that hypermethylation of the *p16INK4a* gene promoter was present in 36% of mucosa samples obtained 10 cm from the tumor in patients with colorectal cancer.^10^ The presence of *p16INK4a* promoter methylation was associated with worse outcomes in the patients. Aberrant methylation at this distance from the tumor may indicate the presence of ubiquitous alterations in the large gut of some patients.

Genome-wide hypomethylation is a frequent epigenetic alteration in carcinomas as well as normal-appearing mucosa of colorectal cancer patients. The long interspersed nucleotide element 1 (LINE-1), which is the most abundant transposable element in the human genome, can be used as a surrogate marker for the global hypomethylation status.^11^ LINE-1 hypomethylation seems to contribute to a “field defect” in dysplastic and premalignant mucosa that influences the progression of colorectal carcinogenesis.^12^

The aim of study was to quantify and compare the methylation levels of *MLH1*, *MGMT*, *p16INK4a*, and LINE-1, and the gene expression levels of *MLH1*, *MGMT*, and *p16INK4a* in colon tumors, matched non-cancerous mucosa and control mucosa to identify early signs of putatively premalignant changes. Methylation and expression data were correlated to tumor MSI and *KRAS* status as well as to demographic and clinicopathological data of patients.

## Patients and methods

### Inclusion of study subjects

Study subjects who underwent colonoscopy at the Sahlgrenska University Hospital, Gothenburg, Sweden were consecutively included in the study. Reasons for referral to colonoscopy for each participant are presented in Additional file 1. Forty controls and 20 patients who were diagnosed with colon cancer were included. Fifteen of the patients (11 women and four men) had tumors located on the right side of the colon whereas five (two women and three men) had tumors on the left side. The prerequisite for inclusion into the control group was ≥18 years of age, and a normal-appearing mucosa in the entire colon, e.g. patients with any significant pathology such as colonic polyps or adenomas, inflammatory bowel disease, malignancy, ischemic colitis etc. were excluded. Possibility of microscopic colitis was ruled out by light microscopic examination of biopsy specimens obtained from the mid-portion of the ascending colon as well as from the sigmoid. Presence of colonic diverticula was accepted provided there were no signs of acute diverticulitis and/or diverticulosis-associated colitis. The prerequisite for inclusion into the colon cancer group was the presence of at least one malignant tumor in the colon and ≥18 years of age. Tumors were classified according to the Tumor – Node – Metastasis (TNM) staging system.^13^

### Collection of tissue samples

GAt colonoscopy, mucosa samples were obtained from the mid-portion of the ascending colon (right side samples) as well from the sigmoid (left side samples) using a regular biopsy forceps. If applicable, a tissue sample was also collected from the tumor. The distance between the tumor and the matching non-cancerous mucosa that was sampled on the same side as the tumor was approximately 10 cm. Tissue samples were frozen immediately in liquid nitrogen and stored at −80°C until used.

### DNA and RNA isolation

Genomic DNA and RNA were isolated from tissue samples using Qiagen AllPrep DNA/RNA/Protein Kit (no. 80004) according to the manufacturer’s instructions. The samples were kept at −20° C until analysis.

### Bisulfite conversion and quantification of methylation levels

Two hundred ng of genomic DNA of each sample were used for bisulfite conversion using EpiTecht® Fast Bisulfite Kit (Qiagen, no. 59802) according to the manufacturer’s protocol. Gene-specific methylation of *MLH1*, *MGMT*, and *p16INK4a* was quantified using pyrosequencing and PyroMark Q24 assays (Qiagen). The regions to analyze were the following: *MLH1*, −209 to −181 from transcription start site; *MGMT*, +17 to + 39 in exon 1; *p16INK4a/+68*, +68 to + 120 in exon 1; and *p16INK4a/+235*, +235 to + 270 in exon 1. Global methylation was quantified using the PyroMark Q24 CpG LINE-1 assay (Qiagen). Information about the assays, including sequence to analyze and number of CpG sites, are presented in Table 1. Assay details and PCR conditions are described in Additional files 2 and 3.

**Table 1.** PyroMark Q24 CpG assays used.

Assay name	Sequence to analyze	Number of CpGs
*MLH1*	YGGATAGYGATTTTTAAYGYGTAAGYGTATA	*5*
*MGMT*	YGTTTTGYGTTTYGAYGTTYGTAGGTTTT	*5*
*p16INK4a/+68*	TYGTTAAGTGTTYGGAGTTAATAGTATTTTTTTYGAGTATTYGTTTAYGGYGT	*6 (5)*^*a*^
*p16INK4a/+235*	GGGTGGGGYGGATYGYGTGYGTTYGGYGGTTGYGGA	*7*
*LINE-1*	TTYGTGGTGYGTYGTTT	*3*

^*a*^The total number of CpG sites varied due to a SNP at the fifth CpG site position (for details see Additional file 8).

### Preparation of samples for pyrosequencing

One μl of Sepharose beads were mixed with 40 μl of binding buffer and 22 μl of water in an Eppendorf tube. Sixty μl of this mix was added to 20 μl of PCR products in a 96 well plate and agitated at 1500 rpm for 10 minutes. The PyroMark Advanced Q24 Plate was filled with 0.375 μM of sequencing primer in 20 μl of annealing buffer. The washes were performed using the vacuum station according to the manufacturer’s instruction. To anneal the samples to sequencing primers, the temperature was increased to 80°C for 5 minutes (*MLH1*) or 2.5 minutes (*MGMT*, *p16INK4a/+68, p16INK4a/+235*, and LINE-1). The samples were then immediately processed in the PyroMark Advanced Q24 instrument (Qiagen).

### Quantification of methylation

Pyrosequencing of the purified single-stranded PCR products and CpG site quantification was accomplished using the PyroMark Q24 and related software (Qiagen). The CpG sites were investigated in both mucosa and tumor samples. Each CpG site was assigned a percentage of methylation by evaluating the C/T ratio. The mean percentage of methylation across the CpG sites was calculated for each sample and each analyzed DNA sequence.

### Preparation of cDNA and real-time quantitative PCR

cDNA was synthesized from total RNA using the High Capacity cDNA Reverse Transcription Kit (ThermoFisher Scientific, no. 4368814) and run on a Bio-Rad T100 Thermal Cycler (Bio-Rad laboratories). The relative gene expression of *MLH1*, *MGMT*, *p16INK4a*, and *CDKN2a* in tumor and mucosa tissue was quantified using TaqMan® Assays labeled with FAM-MGB (ThermoFisher Scientific). Samples were run as duplicates in 96-well plates. Polymerase chain reactions were carried out in 5 μl reactions with 1 × TaqMan™ Gene Expression Master Mix (ThermoFisher Scientific, no. 4369016), 1 × gene-specific assay and 2.5 μl cDNA. The plates were run and analyzed using the Pikoreal qPCR System (ThermoFisher Scientific) according to the manufacturer’s protocol. Thresholds and baselines were set manually, and Ct values were extracted. All Ct values were normalized to the mean of the reference genes *ACTB* and *PPIA* (ΔCt) for each sample. Assay details and cycling conditions are presented in Additional file 4.

### MSI status

The microsatellite status was analyzed using the MSI Analysis System, Version 1.2 (Promega) which includes fluorescently labeled primers for co-amplification of seven markers including five mononucleotide repeat markers (BAT-25, BAT-26, NR-21, NR-24 and MONO-27) and two pentanucleotide repeat markers (Penta C and Penta D). Two ng of DNA was used in a 10 μL reaction volume that contained a fluorophore-labeled primer pair, Taq DNA polymerase, deoxyribonucleotide triphosphate mix and buffer. PCR conditions were as follows: denaturation at 95°C, 11 minutes, then at 96°C for 1 minute, followed by 10 cycles of 94°C for 30 seconds, 58°C for 30 seconds, and 70°C for 1 minute, and 20 cycles at 90°C for 30 seconds, 58°C for 30 seconds, and 70°C for 1 minute, then 60°C for 30 minutes followed by a 4°C soak. After amplification, 9.5 μl deionized formamide were combined with 0.5 μl Internal Lane Standard 600 and 1 μl of the PCR reaction. This mixture was denatured at 95°C for 3 minutes, chilled on ice, and spun briefly in a microcentrifuge. The microsatellite markers were detected on the ABI PRISM® 3730 using PowerPlex 4C Matrix Standard (no. DG4800). Microsatellite instability was defined as peak alterations in the marker electropherogram in the tumor compared with corresponding normal tissue. A tumor was defined as having MSI-H if more than one of the five markers showed instability and as having MSI-L if only one marker showed instability. If no MSI was detected, the tumor was designated MSS. Analysis of the MSI data was done by using Peak scanner Software 2.0 (http://www.appliedbiosystems.com).

### KRAS status

Analysis of KRAS mutations was performed by droplet digital PCR (ddPCR) using the Q×200ddPCR system (Bio-Rad Laboratories) according to the manufacturer’s instructions. Briefly, each reaction consisted of 11 μl ddPCR Supermix, 1 μl of target KRAS-assay and wild type KRAS-assay, respectively (900 nM primers and 250 nM probe), 1 μl restriction enzyme (2 U/μl), 6 μl dH2O, and 2 μl of DNA (15 ng/ul). 20 μl of each reaction was transferred to a droplet generation cartridge, 70 μl of Droplet Generation Oil was added and droplets generated in a Droplet Generator. PCR amplification was performed in a Veriti Thermal Cycler (Life Technologies) with the following conditions: 10 minutes at 95°C, 40 cycles of 30 seconds at 94°C and 60 seconds at 60°C, and 10 minutes at 98°C. After amplification, reactions were stored at 4°C until droplets were read in a Q×200Droplet Reader. A negative control (water) and a positive control were included in each run. Study samples were analyzed in triplicate. Initial data quality control and analysis was performed using QuantaSoft v1.6.6.0320 (Bio-Rad Laboratories).

### Statistical analysis

Baseline methylation values were determined in control mucosa (Additional file 5) and subtracted from values determined in tumors of colon cancer patients before statistical analyses were performed. The data obtained were analyzed by statistical modeling using the commercial software JMP Pro 17.0.0 (SAS Institute). Unless otherwise stated, the data are presented as the mean ± standard deviation (SD) or as the median with a 95% confidence interval. Differences between groups were tested using the Kruskal-Wallis test or Pearson chi-square test. To compare sets of continuous parameters measured in the same sample, the Pearson correlation coefficient (r) was used. *p* values <0.05 were considered significant.

## Results

### Gene-specific hypermethylation in control mucosa

The median methylation levels of *MLH1*, *MGMT*, *p16INK4a/+68*, and *p16INK4a/+235* in mucosa of controls (control mucosa) were 1% (range 0.4–2.4%), 1.8% (range 0.4–10.8), 2.2% (range 0.6–3.4), and 1.7% (range 0.86–5.4), respectively. Out of the total 400 CpG sites analyzed for *MLH1* methylation, only two (0.5%) had a level over 3% (one site 4%, one 5%). The *MGMT* methylation level was 0–5% in the vast majority of CpG sites. However, a higher level (6–13%) was found in 31/400 (7.8%) of the sites. This elevated level was found in all CpG sites on both sides of the colon in four cases, and on the left side in one case (Additional file 6).

The *p16INK4a/+68* and *p16INK4a/+235* methylation levels at individual CpG sites varied from 0–5%. Only 3/400 (0.75%) and 2/560 (0.36%) sites, respectively, had a higher value (6%). There were no significant differences in methylation levels between control mucosa obtained from the right and left side of colon. Representative program of analyzed DNA sequences in a control sample are shown in Additional file 7.

### Gene-specific hypermethylation in non-cancerous mucosa

**Figure 1.**
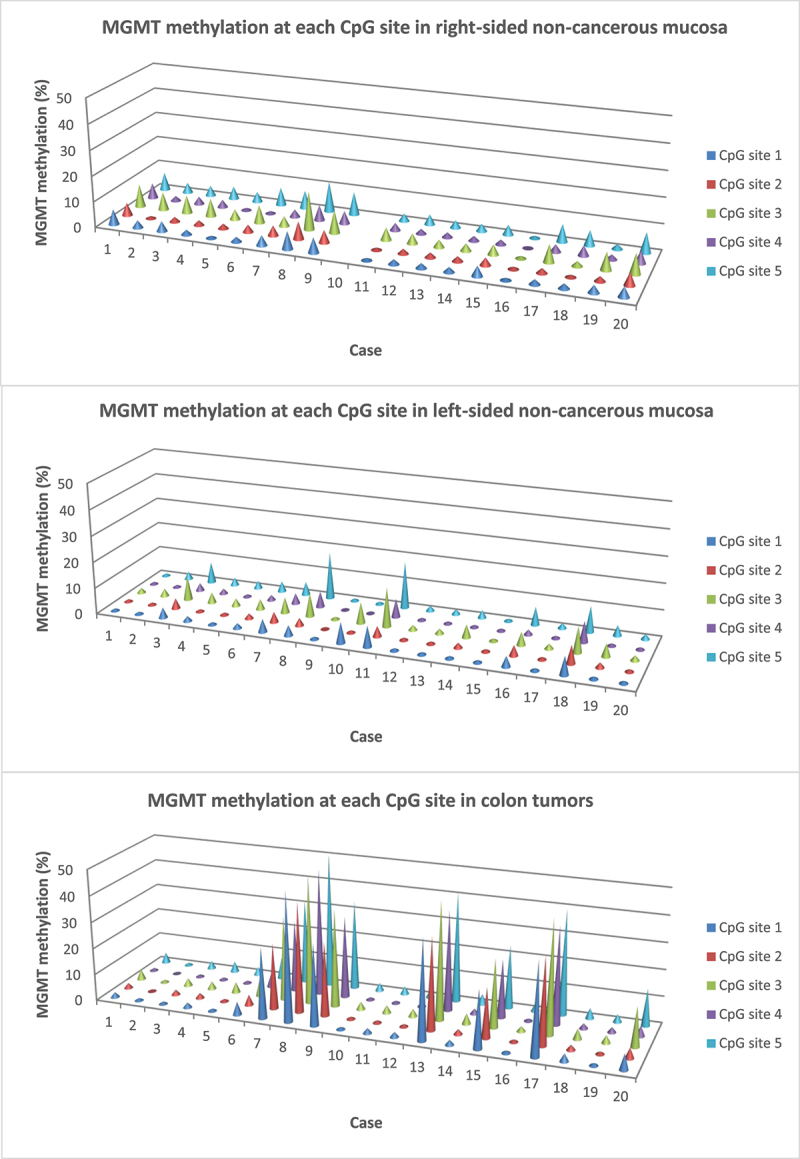
*MGMT* methylation levels at each CpG site in matching (a) right-sided non-cancerous mucosa, (b) left-sided non-cancerous mucosa, and (c) tumor tissue of patients with colon cancer. Case 6, 10, 12, 13, and 14 had a tumor in the left side of colon, all other cases in the right side. No sample could be obtained from right-sided non-cancerous mucosa of case number 10.

The median methylation levels of *MLH1*, *MGMT*, *p16INK4a/+68*, and *p16INK4a/+235* in matching mucosa of colon cancer patients (non-cancerous mucosa) were 1.2% (range 0.6–6.2), 3.0% (range 0.6–10.4), 2.8% (range 1.2–23.6), and 3.6% (range 1.9–7.6), respectively. The level at individual CpG sites varied between cases as well as between sites in one and the same sample. Out of the total 200 CpG sites analyzed for *MLH1* methylation, only seven (3.5%) had a level >5% (range 6–8%). This slightly elevated methylation level was in one case found on both sides of the colon and on the right side in another case. The *MGMT* methylation level was >5% in 17 cases (range 6–18%), only one of which had elevated levels on both sides. The methylation varied greatly, and a value of >5% at all CpG sites was only found in two cases (one right- and one left-sided, Figure 1a,b). In total, 41/200 (20.5%) CpG sites had an MGMT methylation level >5%.

Twenty-three of the 200 (11.5%) CpG sites analyzed for *p16INK4a/+68* had a methylation level > 5% (range 6–40%). Out of five right-sided non-cancerous mucosa samples with a methylation level > 5% at any site, two (40%) had > 5% methylation at all sites, in contrast to only one of seven (14%) left-sided non-cancerous mucosa samples. Sixty-one of the 273 (22.3%) *p16INK4a/+235* CpG sites had a methylation level > 5% (range 6–13%). Seven cases had elevated levels in right- as well as left-sided non-cancerous mucosa, four cases only in the right side, and another four only in the left side. Thus, the methylation level of the four tumor suppressor genes was higher in both right- and left-sided non-cancerous mucosa compared to control mucosa. The differences were significant, as shown in Additional file 5.

### Gene-specific hypermethylation in tumor tissue

In contrast to mucosa samples, the methylation levels at individual CpG sites in the same tumor sample were consistent (Figure 1c). As shown, most cases with *MGMT* methylation in tumors also had a low but elevated (compared to controls) methylation level in non-cancerous mucosa, especially in the right side of colon (*r* = 0.54, *p* = .016, Figure 1a,b). However, increased *MGMT* methylation in non-cancerous mucosa could also be found in cases with no methylation in tumor tissue. High *p16INK4a/+68* methylation was found in both tumor and right-sided non-cancerous mucosa in one case, and several tumors with high *p16INK4a/+235* methylation had an elevated level of methylation in both right- and left-sided non-cancerous mucosa (data not shown).

The methylation levels of the investigated genes in individual tumor samples are presented in Table 2. As shown, 17 tumors had methylation in at least one of the genes whereas the methylation level in three tumors was not above baseline. Two of the tumors showed methylation of all four genes. Methylation of *MLH1* and/or *MGMT* was present in 13 tumors, however, simultaneous methylation of the two genes was only found in three. In contrast, there was a strong, positive correlation between *p16INK4a/+68* and *p16INK4a/+235* methylation (*r* = 0.87, *p* < .0001). A positive correlation was also found between *p16INK4a/+68* and *MLH1* methylation (*r* = 0.45, *p* = .044).

**Table 2.** Methylation levels, MSI status, and KRAS status in individual tumors.

Case	Methylation level (%)					MSI status	*KRAS* G>A mutation
	*MLH1*	*MGMT*	*p16INK4a/+68*	*p16INK4a/+235*	LINE-1		
1	0	0	40.6	25.1	68.3	MSS	Yes
2	0	0	27.0	31.8	70.3	MSS	Yes
3	0	0	35.4	43.2	52.3	MSS	Yes
4	0	0	0	0	75.0	MSS	Yes
5	0	0	0	0	72.7	MSS	Yes
6	0	1.8	4.0	11.5	73.3	MSS	Yes
7	0	24.6	19.4	25.8	65.7	MSS	Yes
8	0	44.0	11.6	35.6	78.0	MSS	Yes
9	0	28.2	1.2	0	79.0	MSS	Yes
10	2.9	0	3.4	0	70.0	MSS	Yes
11	0	0	0	0	67.7	MSS	No
12	0	0	14.2	23.2	80.7	MSS	No
13	0	35.6	0.4	0	80.3	MSS	No
14	3.7	0	0	0	65.3	MSS	No
15	32.3	18.6	30.0	39.2	75.7	MSI-H	No
16	36.7	0	13.0	18.8	73.0	MSI-H	No
17	39.9	33.6	42.8	23.1	60.3	MSI-H	No
18	41.7	0	48.0	43.9	58.0	MSI-H	No
19	57.7	0	49.4	50.6	57.7	MSI-H	No
20	58.7	4.6	0	1.8	58.7	MSI-H	No

### Global methylation in mucosa and tumor tissue

**Figure 2.**
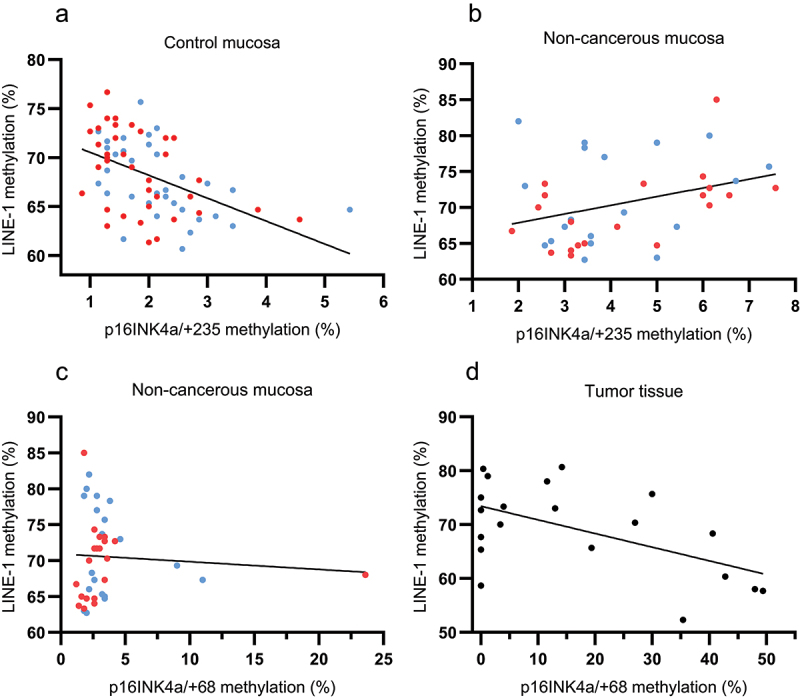
Scatter plots showing (a) a negative correlation (*r* = −0.49, *p* < .0001) between global LINE-1 methylation and *p16INK4a/+235* methylation in control mucosa, (b) a positive correlation (*r* = 0.34, *p* = .036) between LINE-1 and *p16INK4a/+235* methylation in non-cancerous mucosa, (c) lack of correlation between LINE-1 and *p16INK4a/+68* methylation in non-cancerous mucosa, and (d) a negative correlation between LINE-1 methylation and *p16INK4a/+68* methylation in tumor tissue (*r* = −0.55, *p* = .012). Blue dots = right-sided mucosa, red dots = left-sided mucosa.

The median global LINE-1 methylation in control mucosa was 68.8% (range 60.7–76.7) and the level correlated positively between right- and left-sided mucosa (*r* = 0.46, *p* = .024). LINE-1 methylation correlated negatively with both *p16INK4a/+68* and *p16INK4a/+235* methylation in this tissue (*r* = −0.34, *p* = .0017 and *r* = 0.49, *p* < .0001, respectively, Figure 2a).

The LINE-1 methylation level in non-cancerous mucosa (median 69.7%, range 62.7–85.0) was not different from control mucosa (*p* = .1), however, there was no correlation between the levels in right- and left-sided non-cancerous mucosa. Furthermore, LINE-1 methylation correlated positively with *p16INKa/+235* methylation (*r* = 0.34, *p* = .036) whereas there was no correlation with *p16INK4a/+68* (Figure 2b,c). There was also a positive correlation (*r* = 0.47, *p* = .0026) between LINE-1 and *MGMT* methylation in non-cancerous mucosa.

The LINE-1 methylation level in tumors (median 70.2%, range 52.3–80.7) was not significantly different from control or non-cancerous mucosa (*p* = .24). There was a significant negative correlation between LINE-1 and *p16INK4a/+68* methylation (Figure 2d, *r* = −0.55, *p* = .012) as well as *MLH1* methylation (*r* = −0.51, *p* = .021) whereas *p16INK4a/+235* tended to correlate negatively with LINE-1 (*r* = −0.40, *p* = .084).

### Methylation according to MSI and KRAS status of colon cancer patients

Six of the tumors (30%) were found to have high microsatellite instability (MSI-H), whereas 14 (70%) were microsatellite stable (MSS, Table 2). All MSI-H tumors were found in female patients and were located on the right side of colon. Among the MSS cases, six were female, seven were male. Nine MSS tumors were located on the right side, and four on the left side of the colon. As shown in Table 2, all MSI-H cases had a high mean *MLH1* methylation level, ranging from 32% to 58%. In contrast, only two of the MSS tumors had *MLH1* methylation. Although the methylation level in these two tumors was low, it was consistently detected at all five CpG sites. *MGMT* methylation was found in four MSS and two MSI-H tumors. All MSI-H tumors had *p16INK4a/+68* and/or *p16INK4a/+235* methylation. Seven MSS cases had simultaneous *p16INK4a/+68* and *p16INK4a/+235* methylation whereas seven had no or very low methylation (0–7.3%) of any gene. Ten of the tumors (50%) had *KRAS* G > A mutations, all of which were MSS (Table 2). Among KRAS wild-type tumors, 6/10 were MSI-H and 4/10 were MSS. The mean *MLH1* methylation level was significantly lower in *KRAS* mutant tumors (*p* = .0049), but there was no difference in *MGMT* or *p16INK4a* methylation levels according to *KRAS* status.

The mean LINE-1 methylation in MSI-H tumors was 63.9 ± 8.2% versus 71.3 ± 7.6% in MSS tumors (*p* = .11). Out of the six MSI-H tumors, four (67.7%) had a LINE-1 methylation level ≤ 60.3% (Table 2), compared to 1/14 (7.1%) of the MSS tumors. However, the tumor with the lowest LINE-1 methylation, 52.3%, was MSS. There was no difference in LINE-1 methylation according to *KRAS* mutation status.

### Detection and distribution of the p16INK4a rs3814960 variants

Analysis of the *p16INK4a/+68* pyrogram revealed a single nucleotide polymorphism (SNP) at the fifth CpG site (Additional file 8). BLAST analysis showed that this C > T SNP (rs3814960) was located in the 5’-UTR of the *p16INK4a/+68* sequence (Additional file 3). Since the CpG site in the fifth position was only present in tumors having the C variant, this site was not included when the mean methylation percentage was calculated. A search in the National Center for Biotechnology Information database (www.ncbi.nlm.nih.gov) revealed that the T allele frequency is highly variable in different populations having a range from 0.18 in African populations to 0.69 in in Sweden. The distribution of the *p16INK4a* rs3814960 variants differed significantly between controls and colon cancer patients (*p* = .0064, Table 3). Half of the patients had the TT genotype, whereas the CC genotype was only found in two patients. Whereas the genotype frequencies of the patient group were close to the expected, the frequencies of the control group deviated strongly from the expected.

**Table 3.** *p16INK4a* rs3814960 genotype distribution.

	*p16INK4a* rs3814960 genotype		
	CC	CT	TT
Controls, n (%)	9 (22.5)	26 (65)	5 (12.5)
Colon cancer, n (%)	2 (10)	8 (40)	10 (50)

**Figure 3.**
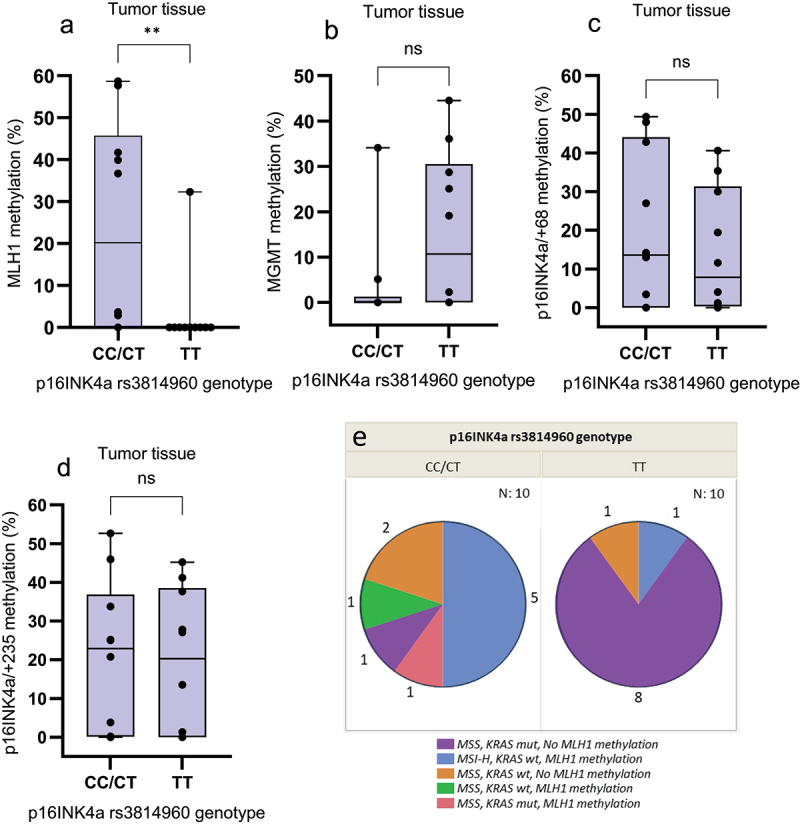
Distribution of (a) *MLH1*, (b) *MGMT*, (c) *p16INK4a/+68*, and (d) *p16INK4a/+235* methylation levels in tumor tissue by *p16INK4a* rs3814960 genotype. The distribution is displayed as box-and-whisker plots with median, 25% and 75% quantiles (box), minimum and maximum (whisker), and outliers. As shown, the methylation level of a) *MLH1* was significantly higher (*p* = .0082) in tumors obtained from patients having the genotype CT/CC compared to TT. b) in contrast, the *MGMT* methylation level in the CT/CC group was lower compared to the TT group, although the difference did not reach significance (*p* = .080). No difference in c) *p16INK4a/+68* or d) *p16INK4a/+235* methylation was seen between the groups (*p* = .68 and 0.91, respectively). (d) The *p16INK4a* rs3814960 TT genotype was associated with tumors which were MSS, KRAS mutant and negative for *MLH1* promoter methylation, whereas the CC/CT genotypes were associated with a mixed group of tumors most of which were *KRAS* wild-type, and positive for *MLH1* methylation.

The methylation level of *MLH1* was significantly higher (*p* = .0082) in tumors of patients having the *p16INK4a* rs3814960 genotype CT/CC compared to TT (Figure 3a). In contrast, the *MGMT* methylation level in the CT/CC group was lower compared to the TT group (Figure 3b), although the difference did not reach significance (0.080). No difference in *p16INK4a/+68* or *p16INK4a/+235* methylation was seen between the groups (*p* = .68 and 0.91, respectively, Figure 3c,d). The mean LINE-1 methylation in TT tumors was non-significantly higher compared to CC/CT tumors (72.0 ± 8.3 vs 66.2 ± 7.6, *p* = .089). Only 1/6 patients (16.7%) with MSI-H tumors had the TT genotype, compared to 9/14 (64.3%) with MSS tumors (*p* = .051). KRAS G>A mutation was found in 8/10 (80%) of the TT tumors, but only in 2/10 (20%) CC/CT tumors (Figure 3e).

### Quantitative gene expression

**Figure 4.**
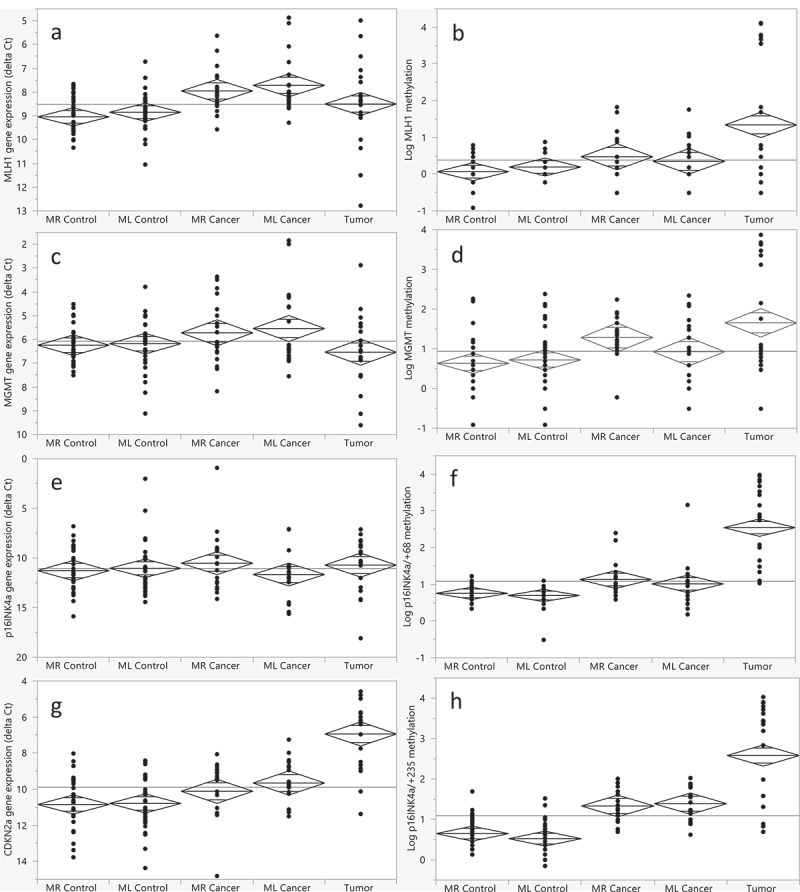
Comparison of *MLH1*, *MGMT*, *p16INK4a*, and *CDKN2a* gene expression (a, c, e, g) and *MLH1*, *MGMT*, *p16INK4a/+68*, and *p16INK4a/+235* methylation (b, d, f, h) in control mucosa (MR/ML control), non-cancerous mucosa (MR/ML cancer), and tumor tissue. MR = right-sided mucosa, ML = left-sided mucosa. Gene expression is presented as ΔCt values. Since a high ΔCt value represents low expression, the scale has been reversed for easier interpretation. The methylation levels (mean percentage in each sample) differed greatly in mucosa and tumors; hence, the values have been logarithmized. The middle line in each mean diamond shows the group mean, whereas the lines above and below the group mean are overlap marks. The top and bottom of the diamonds represents the 95% confidence interval. The horizontal line in each figure shows the grand mean. As shown, the methylation level of each gene was increasingly higher when control mucosa, non-cancerous mucosa, and tumor tissue were compared. In contrast, the expression patterns of the four genes differed. *MLH1* expression in tumors was lower than in matching mucosa but slightly higher compared to control mucosa. The expression of *MGMT* in tumors was lower compared to mucosa of both controls and patients. There was no difference in *p16INK4a* expression, however, *CDKN2a* expression was significantly higher in tumors compared to mucosa.

No significant difference in mean *MLH1*, *MGMT*, *p16INK4a* or *CDKN2a* expression was found between right- and left-sided control mucosa (Figure 4a,c,e,g). There was no correlation between expression of the four genes, with the exception of right-sided control mucosa, where *MGMT* and *p16INK4a* expression correlated positively (*r* = 0.44, *p* = .028). As shown in Figure 4, the expression of *MLH1*, and *CDKN2a* was higher in non-cancerous mucosa compared to control mucosa (*p* < .0001 and *p* = .0011, respectively). However, there was no difference in *MGMT* or *p16INK4a* expression between these two groups (*p* = .16 and *p* = .80, respectively). In right-sided non-cancerous mucosa, the expression of *MGMT* correlated positively with *CDKN2a* expression (*r* = 0.59, *p* = .006).

*MLH1* expression in tumors was also higher compared to control mucosa, however, compared to non-cancerous mucosa it was lower (Figure 4a). *MGMT* expression in tumors was non-significantly lower compared to both control and non-cancerous mucosa (Figure 4c), whereas the expression of *CDKN2a* was significantly higher (*p* < .0001, Figure 4g). No difference in *p16INK4a* expression between mucosa and tumor tissue was seen (Figure 4e). There was a tendency for a correlation between *MGMT* and *p16INK4a* expression (*r* = 0.44, *p* = .066). High methylation of *MLH1* correlated with low *MLH1* expression in tumor tissue (*r* = −0.72, *p* = .0003), and, similarly, high methylation of *p16INK4a/+235* correlated with low *p16INK4a* expression (*r* = −0.50, *p* = .034). However, the correlation between *MGMT* methylation and expression in tumors did not reach significance (*r* = −0.39, *p* = .087).

**Figure 5.**
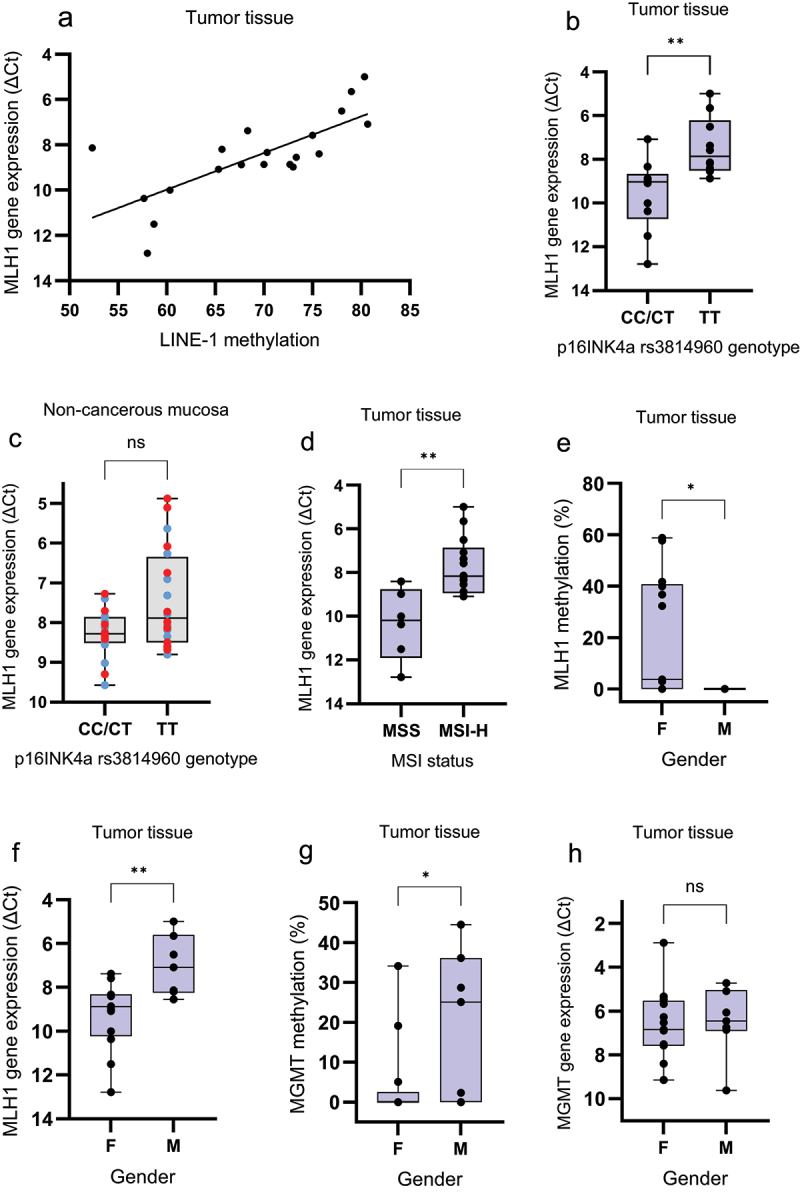
(a) Scatter plot showing a positive correlation (*r* = 0.74, *p* = .0002) between *MLH1* gene expression and LINE-1 methylation in tumor tissue. The gene expression is presented as ΔCt values. Since a high ΔCt value represents low expression, the scale has been reversed for easier interpretation. (b) *MLH1* expression in tumor tissue by *p16INK4a* rs3814960 genotype. (c) *MLH1* expression in non-cancerous mucosa by *p16INK4a* rs3814960 genotype. Blue dots = right-sided mucosa, red dots = left-sided mucosa. (d) *MLH1* expression in tumor tissue by MSI status. The distribution is displayed as box-and-whisker plots with median, 25% and 75% quantiles (box), minimum and maximum (whisker), and outliers. As shown, the *MLH1* expression was non-significantly lower in non-cancerous mucosa (*p* = .076) and significantly) lower (*p* = .0032 in tumor tissue of patients having the *p16INK4a* rs3814960 genotype CC/CT compared to TT, and in patients with MSI-H compared to MSS tumors (*p* = .0034). (e) *MLH1* methylation, (f) *MLH1* gene expression, (g) *MGMT* methylation, and (h) MGMT gene expression in tumor tissue according to gender. The gene expression is presented as ΔCt values. Since a high ΔCt value represents low expression, the scale has been reversed for easier interpretation. The distribution is displayed as box-and-whisker plots with median, 25% and 75% quantiles (box), minimum and maximum (whisker) and outliers. As shown, the methylation level of *MLH1* was significantly higher in females (F) compared to males (M, *p* = .014). In contrast, the *MGMT* methylation level was higher in males, compared to females (*p* = .032). The tumoral expression of *MLH1* was significantly lower in females, compared to males (*p* = .0034) whereas there was no difference between groups regarding *MGMT* expression (*p* = .58).

There was a strong positive correlation between LINE-1 methylation and *MLH1* expression in tumor tissue (*r* = 0.74, *p* = .0002) (Figure 5a), but no correlation between LINE-1 methylation and *MGMT*, *p16INK4a*, or *CDKN2a* expression. The *MLH1* expression was lower in non-cancerous mucosa (*p* = .076) and tumors (*p* = .0032) of patients having the *p16INK4a* rs3814960 genotype CC/CT compared to TT (Figure 5b,c). The *MLH1* expression was significantly lower in tumor tissue of patients with MSI-H compared to MSS tumors (Figure 5d, *p* = .001) as well as in *KRAS* wild-type tumors (*p* = .031). No correlation was found between *MGMT*, *p16INK4a*, or *CDKN2a* expression and the rs3814960 genotype, nor with MSI status. However, 2/10 *KRAS* mutant tumors had rs3814960 genotype CC/CT, compared to 8/10 wild-type tumors (*p* = .0073, Figure 3e).

### Gender differences

There was no difference in gender distribution between controls (14 males, 26 females) and patients (7 males, 13 females). No significant differences in methylation levels were observed in control mucosa according to gender, except for *p16INK4a/+235* which was methylated to a higher level in right-sided mucosa of males (*p* = .012). However, the *p16INK4a* expression did not differ between males and females in right-sided control mucosa. In contrast, *CDKN2a* expression was higher in left side of colon of males compared to females (*p* = .031). No gender-related differences were seen in non-cancerous mucosa, regardless of sampling location.

The methylation level of *MLH1* was higher (*p* = .014) in tumors of females, compared to males (Figure 5e). The higher *MLH1* methylation level was reflected in a lower *MLH1* gene expression in females (*p* = .0034, Figure 5f). In contrast, *MGMT* methylation was lower (*p* = .032) in tumors of females compared to males (Figure 5g), but this was not reflected in *MGMT* expression (Figure 5h). No gender differences were seen according to *p16INK4a/+68* or *p16INK4a/+235* methylation in tumors, nor to *p16INK4a* expression. However, the *p16INK4a* rs3814960 CT/TT genotype was more common among females than males; 9 of 13 women (69.2%) had the CT/TT genotype compared to one of seven men (14.3%, *p* = .019). There was no difference in LINE-1 methylation in control or non-cancerous mucosa according to gender. However, there was a trend for lower LINE-1 methylation in tumors of female patients; the level was 67.1 ± 6.5% in females versus 72.8 ± 10.4% in males (*p* = .081).

### Age correlations

The age of controls, (median 62.5 years, range 33–85), was significantly lower than the age of the patients (median 75 years, range 47–82, *p* = .0066). The methylation level of the analyzed genes in control and non-cancerous mucosa did not correlate significantly with age, nor did gene expression. No correlation between methylation in tumor and age of the colon cancer patients was found when all cases were included. However, a significant positive correlation between *MLH1* methylation and age was seen if only cases with a methylation level above baseline were included (*r* = 0.95, *p* = .0003, *n* = 8). These cases were all females. There was no correlation between *MLH1*, *MGMT*, *p16INK4a*, or *CDKN2a* expression in tumors and age. LINE-1 methylation did not correlate with age in control mucosa. In non-cancerous mucosa, however, there was a positive correlation (*r* = 0.43, *p* = .0051). No correlation between LINE-1 methylation and age was seen in tumor samples.

## Discussion

The physiological conditions associated with aberrant *MLH1* and *MGMT* promoter methylation in colorectal mucosa from healthy individuals undergoing screening colonoscopy were previously analyzed by Menigatti et al..^14^ The authors concluded that the epigenetic signatures of cancers may have early-stage, normal-tissue counterparts that reflect potentially important aspects of the initial carcinogenic process. The results of the present study showed no *MLH1* promoter methylation in control mucosa, however, methylation of CpG sites in exon 1 of *MGMT* varied greatly and was elevated in some individuals supporting the hypothesis. Similar results were found by Shen et al.,^15^ who detected *MGMT* promoter hypermethylation in 12% of the colorectal control mucosa. In the same study, a substantial number of colorectal cancer patients showed *MGMT* methylation in both macroscopically normal mucosa and matching carcinomas. The authors suggested that some colorectal cancers may arise from a field defect defined by epigenetic inactivation of *MGMT*.

Also in the present study, some colon cancer cases showed *MGMT* methylation in both mucosa (especially right-sided) and corresponding tumor. In fact, a significantly higher level of methylation of each analyzed gene was found in mucosa of patients compared with controls. These results are in line with previous findings of our group,^10^ as well as others,^9,16–18^ showing hypermethylation of tumor suppressor genes in mucosa of colorectal cancer patients. For example, Ramirez et al.^9^ examined promoter methylation profiles of *MLH1*, *MGMT*, and *p16INK4a* and concluded that epigenetic changes in mucosa surrounding neoplastic lesions may occur before genetic alterations in early stages of colorectal carcinogenesis. Furthermore, Bihl et al.^17^ analyzed *CDKN2a* methylation in colorectal tumors and matched non-neoplastic tissue, and found that methylation in mucosa ranged from 0 to > 90%. The authors concluded that the non-negligible *CDKN2a* methylation in matching mucosa may confound the assessment of tumor-specific hypermethylation, suggesting that matching mucosa should be used as a control.

In the present study, the methylation level at different CpG sites in both control and non-cancerous mucosa varied considerably compared with tumors, where the level at individual CpG sites was more consistent. The low-grade methylation in mucosa may possibly indicate early dysplastic or premalignant changes. It was also noted that the expression of *MLH1* was lower in tumors compared to matching non-cancerous mucosa but higher compared to the control mucosa. Thus, different conclusions may be drawn depending on whether the tumor is being compared to non-cancerous mucosa or control mucosa. The results suggest that mucosa deriving from healthy individuals is a better control than matching mucosa of cancer patients. The presence of aberrant methylation in control and non-cancerous mucosa could be important in itself since it may represent changes associated with risk of cancer development. Methylation in matching mucosa of colorectal cancer patients may also be used as a predictor of survival as has been shown previously.^10,19,20^

Some previous studies have shown that hypermethylation of *MLH1* and *MGMT* in tumor tissue are mutually exclusive, indicating that the two genes affect different hypermethylation-associated pathways during cancer development.^5^ Whereas *MLH1* methylation is associated with a colorectal cancer molecular subtype that includes CIMP^+^, MSI, and mutations in *BRAF* (but not in *KRAS*), the *MGMT* methylation seems to cross-section the major molecular subtypes. The result of this study favors the hypothesis that the two genes are involved in different pathways; only three out of 13 tumors (23%) had simultaneous methylation of *MLH1* and *MGMT*, and all tumors with MLH1 methylation were MSI-H and lacked *KRAS* G>A mutations. In contrast, 50% of tumors with *MGMT* methylation were *KRAS* mutant. The opposite relationship between *MLH1* and *MGMT* was also seen in relation to other covariates, e.g. gender. *MLH1* methylation was higher in tumors of females compared to males whereas *MGMT* methylation was higher in tumors of males. However, both genes were upregulated in non-cancerous mucosa compared to control mucosa, indicating an increased need for mismatch repair in the tumor surroundings.

Two different assays were used to quantify methylation of a total of 12 CpG sites in exon 1 of *p16INK4a*. Since both assays covered the same exon, it was not unexpected to find a strong correlation between the methylation in the analyzed sequences. In contrast to *MLH1* and *MGMT* expression, the *p16INK4a* expression was consistently low in control and non-cancerous mucosa, as well as in tumor tissue. This finding was unexpected since it did not fit into any of the two patterns described for expression of *p16INK4a* in colon.^21^ No significant correlation was found between *p16INK4a/+68* methylation and expression which is in agreement with a previous study by.^22^ However, there was a negative correlation between *p16INK4a/+235* methylation and expression, indicating that the location of the methylated CpG sites is important for regulation of expression. Interestingly, when using an assay targeting all *CDKN2a* transcripts, i.e. both *p16INK4a* and *p14ARF* mRNA, the expression levels increased in a stepwise fashion when comparing control mucosa, non-cancerous mucosa and tumor tissue. Thus, the *CDKN2a* expression pattern was similar to the stepwise increase in *p16INK4a* expression reported in previous studies,^21,23–25^ and followed the methylation levels of both *p16INK4a/+68* and *p16INK4a/+235*.

When the *p16INK4a/+68* sequence was analyzed, the rs3814960 C>T SNP was identified at the fifth CpG site. Although the genotype frequencies of the patient group were close to the expected, the frequencies of the control group deviated strongly. Only 12.5% had the TT genotype compared to 50% of the patients. At present, it is not known if the deviation in the control group was caused by chance alone, or if it was related to any selection bias. No association was found between the rs3814960 SNP and methylation or expression levels of *p16INK4a*. This is in agreement with a study on patients with lung adenocarcinomas.^26^ However, the methylation level of *MLH1* was significantly lower in tumors with the TT, compared to the CT/CC, genotype. Furthermore, only 16.7% of MSI-H tumors had the TT genotype, compared to 64.3% of the MSS tumors, and all TT tumors were *KRAS* mutant compared to 20% of CT/CC tumors. In addition, global LINE-1 methylation tended to be higher in tumors with the TT genotype. These results suggest an association between the rs3814960 variants and the colorectal cancer molecular subtypes. There are few reports on rs3814960, and at present it is not known if it has any functional impact on colon cancer risk or outcome. However, an association between the TT genotype and better outcome of patients with esophageal squamous carcinomas has been reported.^27^ Furthermore, Buas et al. have shown that the rs3814960 variant lies within a probable 14-bp binding site for the transcription factor early growth response (EGR1), which implies that the SNP may have a functional potential.^28^

It is known that methylation of the promoter region of tumor suppressor genes occurs more frequently in females than males, and in mucosa and tumor tissue located in the right, compared to the left side of the colon.^29^ In addition to higher methylation levels, the right side also has a higher frequency of MSI and lymphocyte infiltration.^30^ The reason for these differences is not clear, but it is known that there are major differences in the etiology of right- and left-sided colon cancers.^31^ Thus, the fact that most of the patients in the present study were females and had tumors located in the right side of colon may explain why the MSI-H frequency in the patient cohort was higher (30%) than the expected 10–15%.

The colon cancer risk increases exponentially with age, and older age and inadequate folate intake are strongly implicated as important risk factors for colon cancer.^32^ The effects of aging and dietary folate on specific features of DNA methylation in the colon of mice have been studied by Keyes et al.^33^ Aging decreased genomic DNA methylation and increased promoter methylation and expression of *CDKN2a*. This effect was however dependent on the level of dietary folate. LINE-1 hypomethylation might also be associated with folate levels in colon tissues. In previous studies, high folate intake correlated with high LINE-1 methylation^34^ whereas low folate correlated with both LINE-1 hypomethylation and gene-specific hypermethylation.^18,35^ In the present study, an inverse correlation was found between LINE-1 methylation and methylation of both *MLH1* and *p16INK4a* which may be related to tissue folate levels. No correlation between the methylation level in tumor and age of patients was found when all cases were included. However, a strong, positive correlation between *MLH1* methylation and age was seen if only those cases with a methylation level above baseline were included. These cases were all females, indicating that the age correlation is gender dependent, as has been suggested by Menigatti et al.^14^ In that study, the prevalence of *MLH1* methylation in normal mucosa increased with age, particularly in right-sided colon of females, whereas samples from males showed no consistent patterns for the promoter.

The low number of patients was a limitation of the study. However, the frequency of tumors with *MLH1*, *MGMT*, and *p16INK4a* hypermethylation agreed with previously published reports, indicating that the patients constituted a representative colon cancer cohort with regard to methylation profiles. Another limitation was the lack of information on methylation and expression of *p14ARF*. The expression of *p14ARF* seems to be related to methylation in exon 1 of *p16INK4a* suggesting an interesting interaction between the two genes that deserves further investigation. It would also be of value to analyze CpG site methylation in exon 2 of *p16INK4a* which, according to a recent report, may influence *p16INK4a* expression.^8^ Furthermore, the SNP (rs16906252 C>T) within the transcriptional enhancer element of the *MGMT* promoter, which recently has been reported to affect the expression of this gene, is of great interest. The T allele entailed reduced transcription in colorectal mucosa of both cases and controls and was associated with an elevated risk of *MGMT*-methylated colorectal cancer.^16,36^ Importantly, beyond serving as a marker of global DNA methylation, LINE-1 itself plays a significant role as a cancer driver, similar to other transposable elements (TE) such as Alu and HERVs.^37,38^ Comparing the expression levels of these elements between matched non-cancerous mucosa and tumor tissue would thus be highly informative. Moreover, given that specific histone modifications, such as H3K9me3, alongside proper DNA methylation, are crucial for maintaining the silencing of TE,^39^ investigating the influence of these epigenetic mechanisms on TE expression would provide valuable insights.

## Conclusions

Using the sensitive method of pyrosequencing, it was possible to analyze and quantify low level methylation at individual CpG sites in mucosa of controls and patients with colon cancer. The methylation levels of tumor suppressor genes in both control and non-cancerous mucosa might be used as early risk markers for carcinogenesis. Control mucosa seems to be a better reference than non-cancerous mucosa when evaluating methylation and expression levels in tumors, since mucosa adjacent to the tumor may have aberrant levels. Although hypermethylation of tumor suppressor genes are common in right-sided colon tumors, no apparent differences in gene-specific methylation, global methylation or gene expression between left- and right-sided colon mucosa were seen, neither in controls nor in patients. The significant association between the *p16INK4a* variant rs3814960 and molecular subgroups of patients with colon cancer is intriguing and deserves to be analyzed in a larger group of patients and controls to establish the normal distribution of the genotypes and any possible impact on colon cancer risk and patient outcome.

## Supplementary Material

Additional_file_4.docx

Additional_file_8.docx

Additional_file_5.docx

Additional_file_1.docx

Additional_file_7.docx

Additional_file_2.docx

Additional_file_6.docx

Additional_file_3.docx

## Data Availability

The datasets used and/or analyzed during the current study are available from the corresponding author on reasonable request.

## Abbreviations

BRAF: B-Raf proto-oncogene, serine/threonine kinase
CDKN2a/p14ARF: Cyclin-dependent kinase inhibitor 2a/p14 alternative open reading frame
CDKN2b/p15INK4b: Cyclin-dependent kinase inhibitor 2b/p15, inhibitor of CDK4/6
CDKN2a/p16INK4a: Cyclin-dependent kinase inhibitor 2a/p16, inhibitor of CDK4/6
CIMP^+^: CpG island methylator phenotype
ddPCR: droplet digital PCR
EGR-1: Early growth response 1
KRAS: Kirsten ras proto-oncogene, GTPase
LINE-1: Long interspersed element 1
MAF: Minor allele frequency
MLH1: MutL Homolog 1
MGMT: O^6^-methylguanine-DNA methyltransferase
MSI-H: Microsatellite instability high
MSS: Microsatellite stable
PCR: Polymerase chain reaction
SNP: Single nucleotide polymorphism
5’-UTR: 5’-untranslated region
