# Supplementary material for: Increased MLH1, MGMT, and p16INK4a methylation levels in colon mucosa potentially useful as early risk marker of colon cancer
Source: Mol Cell Oncol. 2025 May 10;12(1):2503069. doi: 10.1080/23723556.2025.2503069 (PMC12068326; doi:10.1080/23723556.2025.2503069)
Supplement: Additional_file_4.docx [file KMCO_A_2503069_SM8960.docx]

| **Additional file 4. Gene expression assay details.** | | | | | |
| --- | --- | --- | --- | --- | --- |
| Gene | RefSeq | Assay | Exon boundary^a^ | Assay location^b^ | Amplicon length |
| *MLH1* | NM_000249.3 | Hs00979919_m1 | 13/14 | 1757 | 104 |
| *MGMT* | NM_002412.4 | Hs01037698_m1 | 4/5 | 544 | 71 |
| *p16INK4a* | NM_000077.4 | Hs02902543_mH | 1/2 | 454 | 59 |
| *CDKN2a* | NM_000077.4 | Hs00923894_m1 | 2/3 | 764 | 115 |
| *ACTB* | NM_001101.3 | Hs01060665_g1 | 2/3 | 208 | 63 |
| *PPIA* | NM_001300981.1 | Hs99999904_m1 | 5 | 596 | 98 |
| ^a^Probe sits on this boundary.  ^b^Base position contained within the probe. | | | | | |

**PCR conditions**

Denaturation at 95°C for 10 minutes, followed by 40 cycles of denaturation at 95°C for 15 seconds and annealing at 60°C for 60 seconds.
