# Supplementary material for: Increased MLH1, MGMT, and p16INK4a methylation levels in colon mucosa potentially useful as early risk marker of colon cancer
Source: Mol Cell Oncol. 2025 May 10;12(1):2503069. doi: 10.1080/23723556.2025.2503069 (PMC12068326; doi:10.1080/23723556.2025.2503069)
Supplement: Additional_file_8.docx [file KMCO_A_2503069_SM8959.docx]

**
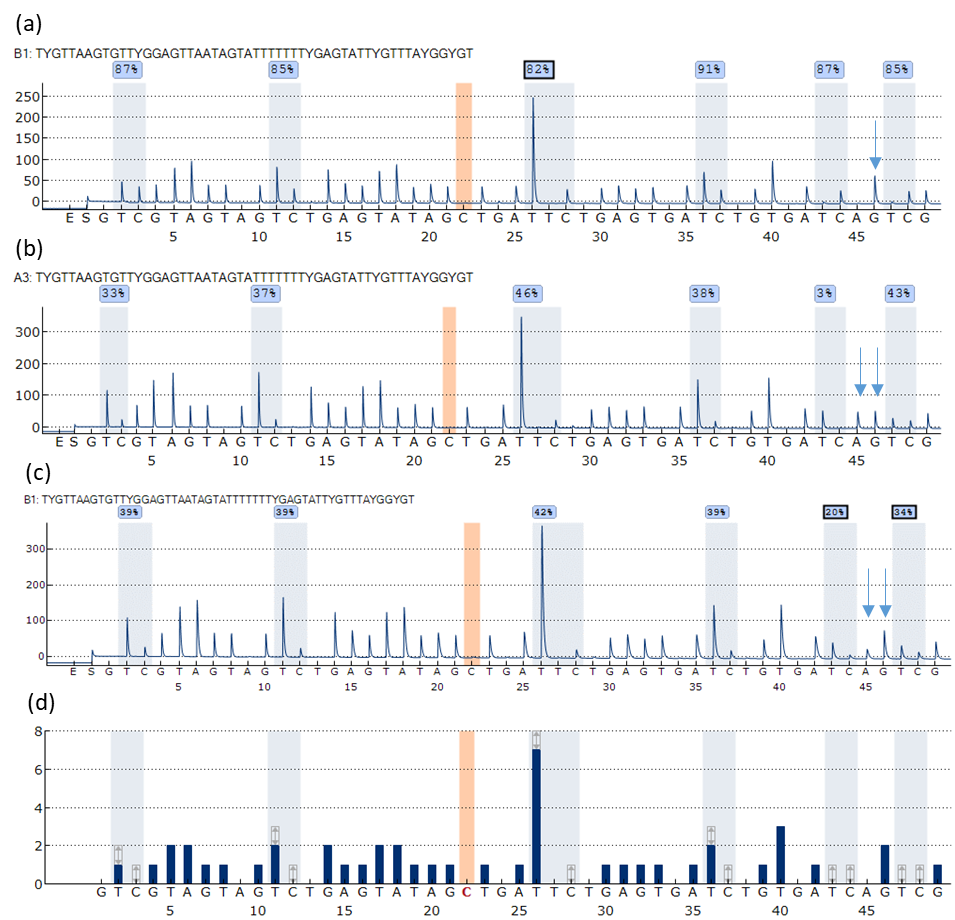
**

**Additional file 8.** Pyrograms showing the effect of the SNP on CpG site 5 of the *p16INK4a/+68* sequence. The sequence before bisulfite conversion was thought to be: TCG…..CAC**G**GCGT with the underlined nucleotides showing CpG site 5. The variant nucleotide is highlighted in red. However, after analysis it was clear that CpG site 5 contained a SNP and that the sequence actually was TCG…..CAC**A/G**GCGT. (a) A true CpG site 5 is seen when both alleles are CC, resulting in a double G peak at position 46 (arrow). (b) If both alleles are TT, there is no methylation above baseline level in CpG site 5, and single peaks of equal heights at positions 45 and 46 are seen (arrows). (c) If there is one C and one T allele present, only the C allele will result in a CpG site in position 5, and a lower methylation level can be seen at this site compared to the surrounding CpG sites. In this case, a low peak can be seen at position 45, and a higher at position 46 (arrows). (d) Histogram for the original *p16INK4a/+68* assay. Controls for completion of bisulfite treatment are highlighted in orange.
