# Supplementary material for: Increased MLH1, MGMT, and p16INK4a methylation levels in colon mucosa potentially useful as early risk marker of colon cancer
Source: Mol Cell Oncol. 2025 May 10;12(1):2503069. doi: 10.1080/23723556.2025.2503069 (PMC12068326; doi:10.1080/23723556.2025.2503069)
Supplement: Additional_file_5.docx [file KMCO_A_2503069_SM8958.docx]

a

b

c

d

Non-cancerous mucosa

Non-cancerous mucosa

Non-cancerous mucosa

Non-cancerous mucosa

Control mucosa

Control mucosa

Control mucosa

Control mucosa

**Additional file 5.** Comparison of mean (a) *MLH1*, (b) *MGMT*, (c) *p16INK4a/+68*, and (d) *p16INK4a/+235* methylation in right- and left-sided mucosa of controls and patients with colon cancer (blue dots = right side, red dots = left side). The methylation levels of *MLH1*, *MGMT*, *p16INK4a/+68,* and *p16INK4a/+235* were significantly higher in non-cancerous mucosa of patients compared to mucosa of controls (p = 0.015, p = 0.0012, p<0.0001, and p<0.0001, respectively). The middle line in each mean diamond shows the group mean, whereas the lines above and below the group mean are overlap marks. The top and bottom of the diamonds represents the 95% confidence interval. The horizontal lines in each diagram correspond to the baseline methylation values in controls used as cut-off values for hypermethylation in patient samples. For *MLH1*, *p16INK4a/+68*, and *p16INK4a/+235,* the cut-off values were set at 2.5%, 4.0, and 5.5%, respectively, that is, at the highest values found in control samples. The cut-off values for *MGMT* was set at 4.0%, excluding the outliers.
