# Supplementary material for: Increased MLH1, MGMT, and p16INK4a methylation levels in colon mucosa potentially useful as early risk marker of colon cancer
Source: Mol Cell Oncol. 2025 May 10;12(1):2503069. doi: 10.1080/23723556.2025.2503069 (PMC12068326; doi:10.1080/23723556.2025.2503069)
Supplement: Additional_file_1.docx [file KMCO_A_2503069_SM8957.docx]

| **Additional file 1.** Reason for referral of study participants to colonoscopy | | | |
| --- | --- | --- | --- |
| **Controls** | | **Patients with colon cancer** | |
| **Case** | **Reason referral** | **Case** | **Reason referral** |
| 1 | Iron deficiency, anemia | 1 | Fatigue, low Hb, positive F-Hb |
| 2 | Obstipation | 2 | Iron deficiency, anemia |
| 3 | Functional diarrhea | 3 | Rectal bleeding, low Hb |
| 4 | IBS | 4 | Anemia |
| 5 | Rectal bleeding | 5 | Abdominal pain |
| 6 | Iron deficiency, anemia | 6 | Changed stool habits, rectal bleeding |
| 7 | IBS | 7 | Anemia |
| 8 | IBS, obstipation | 8 | Abdominal pain |
| 9 | Iron deficiency, anemia | 9 | Rectal bleeding, low Hb |
| 10 | IBS, diarrhea | 10 | Iron deficiency, anemia |
| 11 | Rectal bleeding | 11 | Rectal bleeding |
| 12 | Iron deficiency, anemia | 12 | Abdominal pain |
| 13 | Follow-up after diverticulitis | 13 | Follow-up after adenoma |
| 14 | IBS, diarrhea | 14 | Anemia, rectal bleeding |
| 15 | Follow-up after diverticulitis | 15 | Rectal bleeding |
| 16 | Iron deficiency, anemia | 16 | Changed stool habits |
| 17 | Rectal bleeding | 17 | Fatigue, anemia |
| 18 | Rectal bleeding | 18 | Rectal bleeding, changed stool habits |
| 19 | Diffuse abdominal pain | 19 | Anemia |
| 20 | Diarrhea-obstipation, rectal bleeding | 20 | Changed stool habits, positive F-Hb |
| 21 | Rectal bleeding |  |  |
| 22 | Diarrhea |  |  |
| 23 | Follow-up after diverticulitis |  |  |
| 24 | Obstipation |  |  |
| 25 | Abdominal pain |  |  |
| 26 | Diarrhea-obstipation |  |  |
| 27 | Bleeding, follow-up after diverticulitis |  |  |
| 28 | Changed stool habits, positive F-Hb |  |  |
| 29 | Rectal bleeding |  |  |
| 30 | Rectal bleeding, colon irritabile |  |  |
| 31 | Follow-up after diverticulitis |  |  |
| 32 | Follow-up after diverticulitis |  |  |
| 33 | Follow-up after diverticulitis |  |  |
| 34 | Follow-up after diverticulitis |  |  |
| 35 | Follow-up after diverticulitis |  |  |
| 36 | Follow-up after diverticulitis |  |  |
| 37 | Abdominal pain, changed stool habits |  |  |
| 38 | Follow-up after diverticulitis |  |  |
| 39 | Iron deficiency, anemia |  |  |
| 40 | Obstipation |  |  |
