# Supplementary material for: Increased MLH1, MGMT, and p16INK4a methylation levels in colon mucosa potentially useful as early risk marker of colon cancer
Source: Mol Cell Oncol. 2025 May 10;12(1):2503069. doi: 10.1080/23723556.2025.2503069 (PMC12068326; doi:10.1080/23723556.2025.2503069)
Supplement: Additional_file_7.docx [file KMCO_A_2503069_SM8956.docx]

**
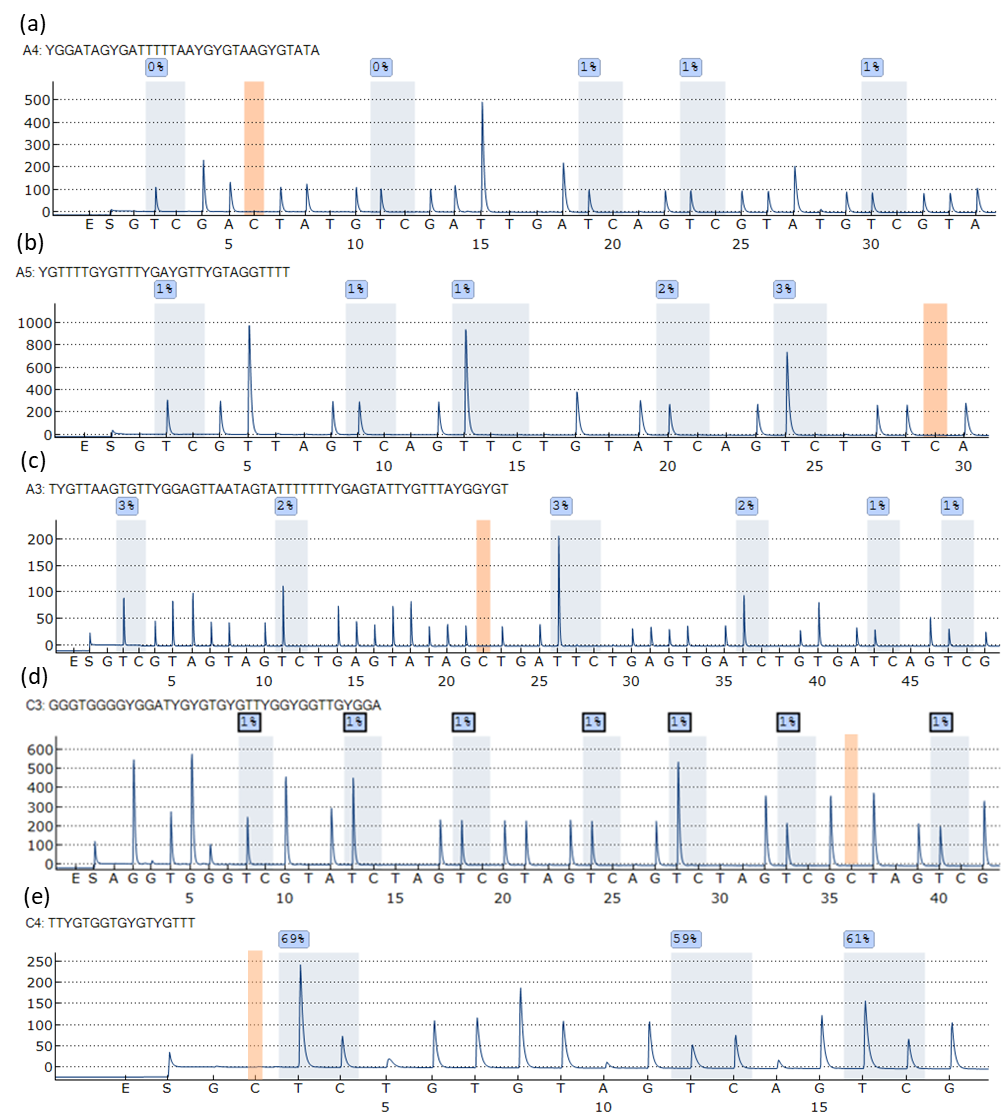
**

**Additional file 7.** Representative pyrograms showing percent methylation at each CpG site in a) *MLH1*, b) *MGMT*, c) *p16INK4a/+68*, d) *p16INK4a/+235*, and e) LINE-1 in control mucosa. Controls for completion of bisulfite treatment are highlighted in orange.
