# Supplementary material for: Increased MLH1, MGMT, and p16INK4a methylation levels in colon mucosa potentially useful as early risk marker of colon cancer
Source: Mol Cell Oncol. 2025 May 10;12(1):2503069. doi: 10.1080/23723556.2025.2503069 (PMC12068326; doi:10.1080/23723556.2025.2503069)
Supplement: Additional_file_2.docx [file KMCO_A_2503069_SM8955.docx]

**Additional file 2**. **Pyrosequencing PCR conditions.**

The PyroMark PCR Kit (Qiagen, Cat No./ID: 978703) was used for the PCR. The Kit includes Coral Load and a PyroMark PCR Master Mix containing HotStarTaq DNA Polymerase, dNTPs, and optimized PyroMark Reaction Buffer with MgCl_2_. The final MgCl_2_ concentration was 3 mM for *MLH1*, 2.25 mM for *MGMT* and *p16INK4a*/*+235*, and 1.5 mM for *p16INK4a/+68* and LINE-1.

| **Methylation detection assay details** | | |
| --- | --- | --- |
| **Gene** | **Assay** | **Cat No./ID.** |
| *MLH1* | PyroMark Q24 CpG MLH1 methylation detection assay | 970022 |
| *MGMT* | PyroMark Q24 CpG MGMT methylation detection assay | 970032 |
| *p16INK4a/+68* | Hs_CDKN2A_02_PM PyroMark CpG Assay | PM00039907 |
| *p16INK4a/+235* | PyroMark Q24 CpG p16 methylation detection assay | 970012 |
| LINE-1 | PyroMark Q24 CpG LINE-1 methylation detection assay | 970042 |

**PCR conditions for *MLH1, p16INK4a/+68* and *p16INK4a/+235***

Denaturation at 95°C for 15 minutes, followed by 45 cycles of denaturation at 95°C for 20 seconds, annealing at 55°C for 20 seconds, and extension at 72°C for 20 seconds, then final extension at 72°C for 5 minutes.

**PCR conditions for *MGMT***

Denaturation at 95°C for 15 minutes, followed by 45 cycles of denaturation at 95°C for 20 seconds, annealing at 53°C for 20 seconds, and extension at 72°C for 20 seconds, then final extension at 72°C for 5 minutes.

**PCR conditions for LINE-1**

Denaturation at 95°C for 15 minutes, followed by 45 cycles of denaturation at 94°C for 30 seconds, annealing at 50°C for 30 seconds, and extension at 72°C for 30 seconds, then final extension at 72°C for 10 minutes.
