# Supplementary material for: Increased MLH1, MGMT, and p16INK4a methylation levels in colon mucosa potentially useful as early risk marker of colon cancer
Source: Mol Cell Oncol. 2025 May 10;12(1):2503069. doi: 10.1080/23723556.2025.2503069 (PMC12068326; doi:10.1080/23723556.2025.2503069)
Supplement: Additional_file_6.docx [file KMCO_A_2503069_SM8954.docx]

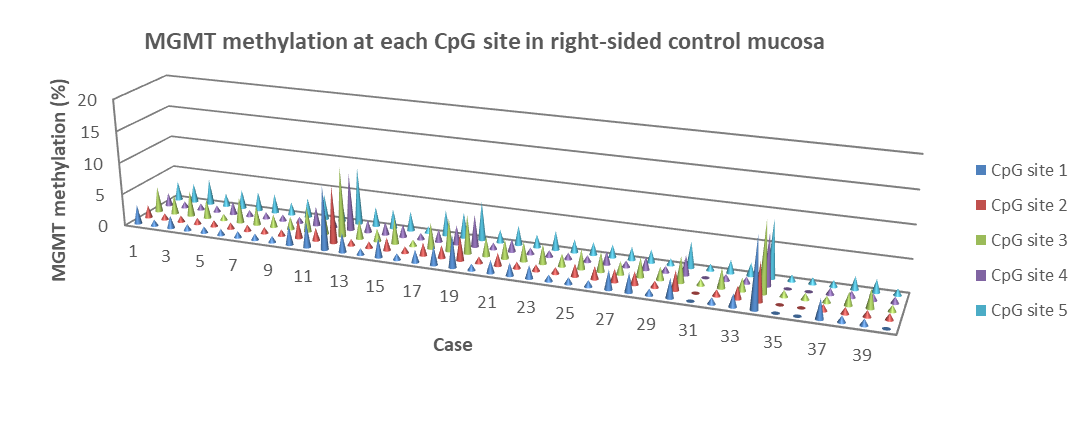

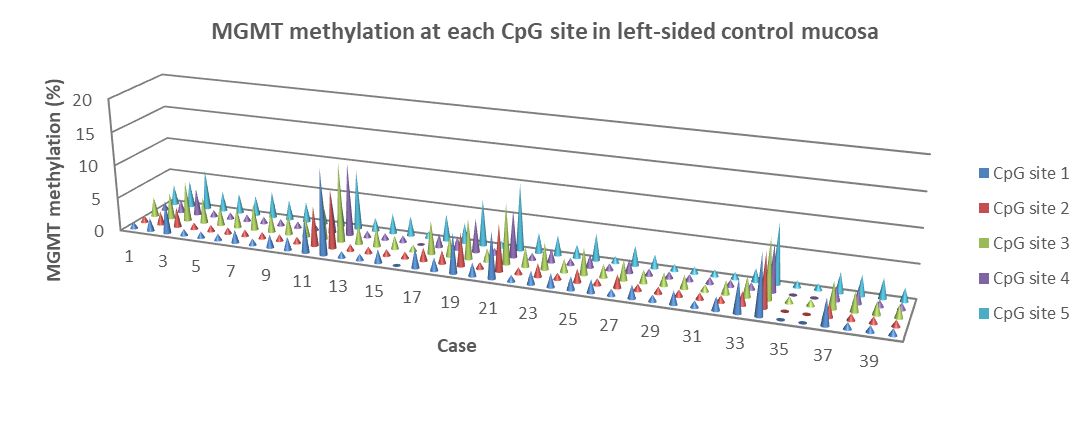

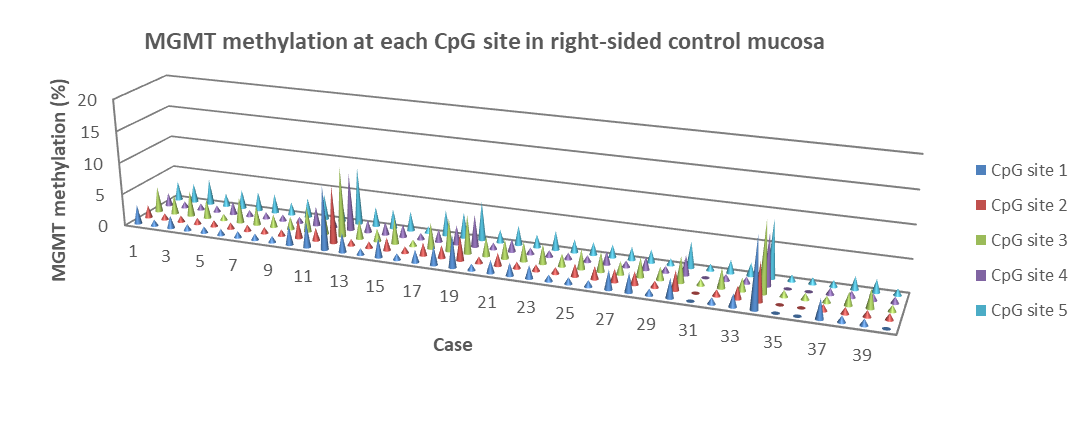

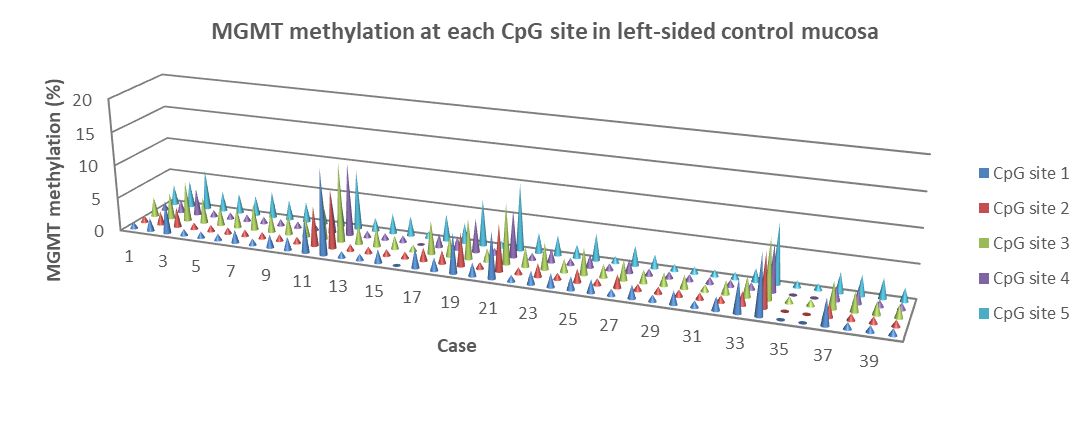


a

b

**Additional file 6.** *MGMT* methylation levels at each CpG site in (a) right-sided control mucosa, and b) left-sided control mucosa. Elevated methylation levels are found on both sides in case 11, 12, 19, and 34, whereas case 21 had an elevated level in mucosa deriving from the left side only.
