# Supplementary material for: Increased MLH1, MGMT, and p16INK4a methylation levels in colon mucosa potentially useful as early risk marker of colon cancer
Source: Mol Cell Oncol. 2025 May 10;12(1):2503069. doi: 10.1080/23723556.2025.2503069 (PMC12068326; doi:10.1080/23723556.2025.2503069)
Supplement: Additional_file_3.docx [file KMCO_A_2503069_SM8953.docx]

**Homo sapiens cyclin dependent kinase inhibitor 2A (CDKN2A), transcript variant 1, mRNA**

NCBI Reference Sequence: NM_000077.4

<https://www.ncbi.nlm.nih.gov/nuccore/NM_000077.4>

ORIGIN

        1 cgagggctgc ttccggctgg tgcccccggg ggagacccaa cctggggcga cttcaggggt

       61 gccacat**tcg ctaagtgctc ggagttaata gcacctcctc cgagcactcg ctcaCggcgt**

      121 ccccttgcct ggaaagatac cgcggtccct ccagaggatt tgagggacag ggtcggaggg

      181 ggctcttccg ccagcaccgg aggaagaaag aggaggggct ggctggtcac caga**gggtgg**

      241 **ggcggaccgc gtgcgctcgg cggctgcgga** gagggggaga gcaggcagcg ggcggcgggg

      301 agcagcatgg agccggcggc ggggagcagc atggagcctt cggctgactg gctggccacg

      361 gccgcggccc ggggtcgggt agaggaggtg cgggcgctgc tggaggcggg ggcgctgccc

      421 aacgcaccga atagttacgg tcggaggccg atccaggtca tgatgatggg cagcgcccga

      481 gtggcggagc tgctgctgct ccacggcgcg gagcccaact gcgccgaccc cgccactctc

      541 acccgacccg tgcacgacgc tgcccgggag ggcttcctgg acacgctggt ggtgctgcac

      601 cgggccgggg cgcggctgga cgtgcgcgat gcctggggcc gtctgcccgt ggacctggct

      661 gaggagctgg gccatcgcga tgtcgcacgg tacctgcgcg cggctgcggg gggcaccaga

      721 ggcagtaacc atgcccgcat agatgccgcg gaaggtccct cagacatccc cgattgaaag

      781 aaccagagag gctctgagaa acctcgggaa acttagatca tcagtcaccg aaggtcctac

      841 agggccacaa ctgcccccgc cacaacccac cccgctttcg tagttttcat ttagaaaata

      901 gagcttttaa aaatgtcctg ccttttaacg tagatatatg ccttccccca ctaccgtaaa

      961 tgtccattta tatcattttt tatatattct tataaaaatg taaaaaagaa aaacaccgct

     1021 tctgcctttt cactgtgttg gagttttctg gagtgagcac tcacgcccta agcgcacatt

     1081 catgtgggca tttcttgcga gcctcgcagc ctccggaagc tgtcgacttc atgacaagca

     1141 ttttgtgaac tagggaagct caggggggtt actggcttct cttgagtcac actgctagca

     1201 aatggcagaa ccaaagctca aataaaaata aaataatttt cattcattca ctcaaaaaaa

     1261 aaaaaaa

//

**Additional file 3**. The *CDKN2a* mRNA transcript variant 1α includes exon 1 (marked in gray) which is specific for *p16INK4a*. The sequence analyzed by the Hs_CDKN2a_02_PM PyroMark CpG assay PM00039907 (*p16INK4a/+68*) is located at +68 to +120 in exon 1 (marked in yellow) and detects a SNP (rs3814960) at position +115 (marked with the capital letter “C”). The sequence analyzed by the PyroMark Q24 CpG p16 methylation assay 970012 (*p16INK4a/+235*) is located at +235 to +270 in exon 1 (marked in white).
